# Supplementary material for: Enhanced accuracy and adaptability: An ISSA-optimized MPC approach for AGV trajectory tracking
Source: PLoS One. 2026 May 21;21(5):e0345476. doi: 10.1371/journal.pone.0345476 (PMC13193454; doi:10.1371/journal.pone.0345476)
Supplement: Attachment — Submitted filename: Response to Reviewers.pdf [file pone.0345476.s003.pdf]

Response to Reviewers

Contents

1. Journal Requirements 2
1.1 Response to Requirement 1 2
1.2 Response to Requirement 2 2
1.3 Response to Requirement 3 3
1.4 Response to Requirement 4 3
1.5 Response to Requirement 5 5
1.6 Response to Requirement 6 6
1.7 Response to Requirement 7 7
1.8 Response to Requirement 8 8
2. Review Comments to the Author 9
2.1 Comments from Reviewer #1 9
2.1.1 Response to Comment 1 9
2.1.2 Response to Comment 2 10
2.1.3 Response to Comment 3 10
2.1.4 Response to Comment 4 11
2.1.5 Response to Comment 5 12
2.1.6 Response to Comment 6 12
2.2 Comments from Reviewer #2 13
2.2.1 Response to Comment 1 13
2.2.2 Response to Comment 2 14
2.2.3 Response to Comment 3 15
2.2.4 Response to Comment 4 16
2.2.5 Response to Comment 5 16
2.2.6 Response to Comment 6 17
2.2.7 Response to Comment 7 20
2.2.8 Response to Comment 8 23
2.2.9 Response to Comment 9 25

We sincerely thank the editor and reviewers for their valuable feedback, which has helped us improve the quality of the manuscript. The comments from the editor and reviewers are listed below in ***bold and italicized*** font, and specific issues have been numbered. Our responses are provided in normal font, and the changes/additions to the manuscript are highlighted in blue.

As the paper template does not indicate section numbers, the corresponding section numbers are specified as follows:

0 Introduction

1 Establishment of the AGV motion model

1.1 Kinematic model

1.2 Dynamic model

2 MPC controller design

2.1 Linearization and discretization of nonlinear systems

2.2 Output prediction equations

2.3 Objective function design

2.4 Reformulation as a quadratic programming problem for solution

2.5 Feedback correction

3 Adaptive time-domain MPC controller design

3.1 Impact of time-domain parameters on tracking performance

3.2 Improved sparrow search algorithm

3.2.1 Discovery phase with dynamically adjusted exploration and exploitation

3.2.2 Adaptive mutation in the follower phase

3.2.3 Dynamic boundary constraints in the sentinel phase

3.3 Fitness function selection

3.4 Tent chaotic map

4 Simulation analysis and experimental verification

4.1 Simulation analysis of the double lane change maneuver

4.2 Simulation analysis of the straight-line maneuver

4.3 Forklift-type AGV experimental validation

5 Conclusion

The specific responses to the editor and reviewers' comments are as follows:

1. Journal Requirements

1.1 Response to Requirement 1

Requirement 1：

Please ensure that your manuscript meets PLOS ONE's style requirements, including those for file naming. The PLOS ONE style templates can be found at:

https://journals.plos.org/plosone/s/file?id=wjVg/PLOSOne_formatting_sample_main_body.pdf and https://journals.plos.org/plosone/s/file?id=ba62/PLOSOne_formatting_sample_title_authors_affiliations.pdf

Response 1：

We sincerely thank the editor for the reminder. We have carefully reviewed the formatting requirements of PLOS ONE (including the two templates you provided: the main text formatting guide and the title page formatting guide) and will comprehensively revise the format, file naming, and other aspects of the manuscript to ensure full compliance with the journal's style requirements.

Specifically, the revisions include: ①adding figure legends; ②adjusting the title format; ③increasing the line spacing of the main text; ④formatting the title, author names, and affiliations; ⑤removing funding information from the acknowledgments; ⑥revising the reference format; and ⑦deleting the Data Availability statement.

1.2 Response to Requirement 2

Requirement 2：

Please note that PLOS One has specific guidelines on code sharing for submissions in which author-generated code underpins the findings in the manuscript. In these cases, we expect all author-generated code to be made available without restrictions upon publication of the work.

Please review our guidelines at https://journals.plos.org/plosone/s/materials-and-software-sharing#loc-sharing-code and ensure that your code is shared in a way that follows best practice and facilitates reproducibility and reuse.

Response 2：

Thank you for your valuable suggestion regarding code sharing. We fully understand the importance of sharing code for enhancing research transparency and reproducibility. The source code for this study was developed within a technology transfer project in collaboration with an enterprise, and its ownership is jointly held by both parties. After consultation with the enterprise, due to intellectual property considerations, including pending patents, they have

requested that the code not be publicly released at this stage. In light of these confidentiality requirements, we are currently unable to make the code publicly available. However, once the intellectual property issues are resolved, we would be happy to consider reasonable requests for access to the code for the purpose of verifying the results of this study.

1.3 Response to Requirement 3

Requirement 3：

We note that the grant information you provided in the 'Funding Information' and 'Financial Disclosure' sections do not match.

When you resubmit, please ensure that you provide the correct grant numbers for the awards you received for your study in the 'Funding Information' section.

Response 3：

Thank you for pointing out the inconsistency in our funding information. We have carefully reviewed and corrected the details.

The correct information for both sections is as follows:

Funding information (in the submission system):

Funder 1: Tianjin Science and Technology Plan Project

Grant number 1: 25ZXRGGX00310

Funder 2: Shijiazhuang Science and Technology Cooperation Special Project

Grant number 2: SJZZXA25003

Financial Disclosure statement: "This work was supported by the Tianjin Science and Technology Plan Project (Grant No. 25ZXRGGX00310) and the Shijiazhuang Science and Technology Cooperation Special Project (Grant No. SJZZXA25003)."

Since we could not find the Financial Disclosure option in the submission system, we kindly request that you update the information on our behalf.

Please let us know if any further adjustments are needed.

1.4 Response to Requirement 4

Requirement 4：

Thank you for stating the following in the Acknowledgments Section of your manuscript:

"This work was supported by the Tianjin Science and Technology Plan Project (Grant No. 25ZXRGGX00310) and the Shijiazhuang Science and Technology Cooperation Special Project (Grant No. SJZZXA25003). Thanks to Prof. Chengjun Ding for his guidance. Thanks to Tan Zhang, Jingyu Ge, Tengfei Ma, Zijian Li, Zhikai Jing, Jinshen Yu and Jianing

Zhang for their assistance in the project."

We note that you have provided funding information that is not currently declared in your Funding Statement. However, funding information should not appear in the Acknowledgments section or other areas of your manuscript. We will only publish funding information present in the Funding Statement section of the online submission form.

Please remove any funding-related text from the manuscript and let us know how you would like to update your Funding Statement. Currently, your Funding Statement reads as follows:

"The author(s) received no specific funding for this work."

Please include your amended statements within your cover letter; we will change the online submission form on your behalf.

Response 4：

Thank you for your guidance regarding the placement of funding information. We have revised the manuscript accordingly and would like to update the Funding Statement as follows.

(1) Removal from the Acknowledgments section

We have removed the following funding-related text from the Acknowledgments section of the manuscript:

"This work was supported by the Tianjin Science and Technology Plan Project (Grant No. 25ZXRGGX00310) and the Shijiazhuang Science and Technology Cooperation Special Project (Grant No. SJZZXA25003)."

The Acknowledgments section now contains only personal acknowledgments, as follows:

"Thanks to Prof. Chengjun Ding for his guidance. Thanks to Tan Zhang, Jingyu Ge, Tengfei Ma, Zijian Li, Zhikai Jing, Jinshen Yu and Jianing Zhang for their assistance in the project."

(2) Updated Funding Statement

Please replace the current Funding Statement ("The authors received no specific funding for this work.") with:

"This work was supported by the Tianjin Science and Technology Plan Project (Grant No. 25ZXRGGX00310) and the Shijiazhuang Science and Technology Cooperation Special Project (Grant No. SJZZXA25003)."

We have ensured that no funding information appears elsewhere in the manuscript. As we could not locate the Funding Statement option in the submission system, we kindly request that you update it on our behalf.

Please let us know if any further adjustments are needed.

1.5 Response to Requirement 5

Requirement 5:

We note that your Data Availability Statement is currently as follows:

"All relevant data are within the manuscript and its Supporting Information files."

Please confirm at this time whether or not your submission contains all raw data required to replicate the results of your study. Authors must share the "minimal data set" for their submission. PLOS defines the minimal data set to consist of the data required to replicate all study findings reported in the article, as well as related metadata and methods (https://journals.plos.org/plosone/s/data-availability#loc-minimal-data-set-definition).

For example, authors should submit the following data:

- The values behind the means, standard deviations and other measures reported;
- The values used to build graphs;
- The points extracted from images for analysis.

Authors do not need to submit their entire data set if only a portion of the data was used in the reported study.

If your submission does not contain these data, please either upload them as Supporting Information files or deposit them to a stable, public repository and provide us with the relevant URLs, DOIs, or accession numbers. For a list of recommended repositories, please see https://journals.plos.org/plosone/s/recommended-repositories.

If there are ethical or legal restrictions on sharing a de-identified data set, please explain them in detail (e.g., data contain potentially sensitive information, data are owned by a third-party organization, etc.) and who has imposed them (e.g., an ethics committee). Please also provide contact information for a data access committee, ethics committee, or other institutional body to which data requests may be sent. If data are owned by a third party, please indicate how others may request data access.

Response 5:

Thank you for your inquiry regarding our Data Availability Statement. We have carefully reviewed PLOS's policy on the minimum dataset.

Regarding shareable data: We have provided all processed data necessary to generate the figures and statistical analyses presented in the manuscript. These data, including the trajectories in the path tracking plots and the error metrics reported in the results, are available in the Supporting Information file (Figures_and_Related_Data.zip). This dataset is sufficient to verify the comparative performance conclusions presented in this study.

Regarding restricted raw data: The complete raw data logs (e.g., high-frequency sensor data streams, internal control signals) are owned by our industrial partner as part of a technology transfer agreement and are subject to a pending patent. Due to these intellectual property and

confidentiality considerations, the full raw dataset cannot be made publicly available.

Updated Data Availability Statement: We have revised the Data Availability Statement to read as follows:

"No - some restrictions will apply."

The TIFF figures and related data have been uploaded to the submission system under the file name: Figures_and_Related_Data.zip.

We believe this approach satisfies the journal's requirements while respecting the necessary intellectual property protections.

1.6 Response to Requirement 6

Requirement 6:

We note that Figure 11 in your submission contain copyrighted images. All PLOS content is published under the Creative Commons Attribution License (CC BY 4.0), which means that the manuscript, images, and Supporting Information files will be freely available online, and any third party is permitted to access, download, copy, distribute, and use these materials in any way, even commercially, with proper attribution. For more information, see our copyright guidelines: http://journals.plos.org/plosone/s/licenses-and-copyright.

We require you to either (1) present written permission from the copyright holder to publish these figures specifically under the CC BY 4.0 license, or (2) remove the figures from your submission:

1) You may seek permission from the original copyright holder of Figure 11 to publish the content specifically under the CC BY 4.0 license.

We recommend that you contact the original copyright holder with the Content Permission Form (http://journals.plos.org/plosone/s/file?id=7c09/content-permission-form.pdf) and the following text:
"I request permission for the open-access journal PLOS ONE to publish XXX under the Creative Commons Attribution License (CCAL) CC BY 4.0 (http://creativecommons.org/licenses/by/4.0/). Please be aware that this license allows unrestricted use and distribution, even commercially, by third parties. Please reply and provide explicit written permission to publish XXX under a CC BY license and complete the attached form."

Please upload the completed Content Permission Form or other proof of granted permissions as an ""Other"" file with your submission.

In the figure caption of the copyrighted figure, please include the following text: "Reprinted from [ref] under a CC BY license, with permission from [name of publisher], original copyright [original copyright year]."

2) If you are unable to obtain permission from the original copyright holder to publish these figures under the CC BY 4.0 license or if the copyright holder's requirements are incompatible with the CC BY 4.0 license, please either i) remove the figure or ii) supply a replacement figure that complies with the CC BY 4.0 license. Please check copyright information on all replacement figures and update the figure caption with source information.

If applicable, please specify in the figure caption text when a figure is similar but not identical to the original image and is therefore for illustrative purposes only.

Response 6:

Thank you for your careful review of Fig. 11. We would like to clarify that this figure is entirely original and created by the authors.

The forklift-type AGV shown in Fig. 11 is a self-developed prototype modified from a traditional electric forklift. The photograph was taken by the authors, and all annotations were manually added. No third-party copyrighted materials are included in this figure.

Therefore, this figure fully complies with the CC BY 4.0 license requirements, and no external copyright permission is needed.

Please let us know if any further documentation or confirmation is required.

1.7 Response to Requirement 7

Requirement 7:

If the reviewer comments include a recommendation to cite specific previously published works, please review and evaluate these publications to determine whether they are relevant and should be cited. There is no requirement to cite these works unless the editor has indicated otherwise.

Response 7:

Thank you for your attention. We have evaluated the literature recommended by the reviewer and related studies, and carefully reviewed the suggested references, confirming that they are highly relevant to this work. Therefore, we have incorporated them into the revised manuscript.

Specifically, the following references have been added to the Introduction section to support the literature review on adaptive MPC and trajectory tracking control (Page 2):

25. Jinhao Liang, Qingyun Tian, Jiwei Feng, Dawei Pi, Guodong Yin. A Polytopic Model-Based Robust Predictive Control Scheme for Path Tracking of Autonomous Vehicles. IEEE Transactions on Intelligent Vehicles. 2024; 9(2): 3928-3939. DOI: 10.1109/TIV.2023.3340668

26. Jinhao Liang, Yanbo Lu, Faan Wang, Jiwei Feng, Dawei Pi, Guodong Yin. ETS-

Based Human–Machine Robust Shared Control Design Considering the Network Delays. IEEE Transactions on Automation Science and Engineering. 2025; 22: 17501-17511. DOI: 10.1109/TASE.2024.3383094

35. Qi Xia, Peng Chen, Guoyan Xu, Bin Zhou, Guizhen Yu. Reinforcement Learning-Based Adaptive Safety Tracking Controller for Autonomous Mining Trucks. IEEE Transactions on Vehicular Technology. 2025; 74(7): 10122-10136. DOI: 10.1109/TVT.2025.3546647

36. Kairui Chen, Yixiang Gu, Weicong Huang, Zhonglin Zhang, Zian Wang, Xiaofeng Wang. Fixed-Time Adaptive Event-Triggered Guaranteed Performance Tracking Control of Nonholonomic Mobile Robots under Asymmetric State Constraints. Mathematics. 2024; 12(10): 1471. https://doi.org/10.3390/math12101471

37. Huarong Zheng, Rudy R. Negenborn, Gabriël Lodewijks. Fast ADMM for Distributed Model Predictive Control of Cooperative Waterborne AGVs. IEEE Transactions on Control Systems Technology. 2017; 25(4): 1406-1413. DOI: 10.1109/TCST.2016.2599485

These references have been cited in the Introduction to discuss model-based adaptive methods [25,26], modern optimization approaches [35,36], and distributed MPC frameworks [37], respectively. We believe they enrich the literature review and help better position the proposed ISSA-MPC framework within the current research context.

1.8 Response to Requirement 8

Requirement 7:

We've checked your submission and before we can proceed, we need you to address the following issues:

(1) Please amend your list of authors on the manuscript to ensure that each author is linked to an affiliation.

(2) Please ensure that you refer to Table 3 in your text as, if accepted, production will need this reference to link the reader to the Table.

Response 7:

Thank you for your careful reminders.

(1) Regarding the author affiliation issue, we have added numeric superscripts next to each author's name on the title page, and each superscript corresponds to the author's affiliated institution.

(2) Regarding the citation of Table 3, we have added “As shown in Table 3” in the text (Section 4.3, page 21, the paragraph just below Table 3) to ensure that Table 3 is properly cited.

2. Review Comments to the Author

2.1 Comments from Reviewer #1

2.1.1 Response to Comment 1

Comment 1：

The core contribution of the manuscript lies in improving the Sparrow Search Algorithm and using it to optimize MPC parameters online. However, the paper mainly introduces several heuristic modifications, including Tent chaotic initialization, disturbance factors, and Cauchy mutation, without clearly explaining the theoretical motivation behind these design choices. It would strengthen the manuscript if the authors could more clearly explain why these specific mechanisms improve the search behavior of the algorithm and how they address the limitations of the standard SSA in the context of MPC parameter tuning.

Response 1：

We sincerely thank the reviewer for this insightful comment. We agree that the theoretical motivation behind the improvement mechanisms should be more clearly articulated. In the revised manuscript, we have added detailed explanations in Section 3.2 to clarify why each mechanism is specifically suited to address the limitations of the standard SSA in the context of MPC parameter tuning.

The key additions are summarized as follows:

Tent chaotic initialization (Page 11, Section 3.2): The standard SSA uses random initialization, which often leads to uneven population distribution and limits global search capability. In MPC parameter tuning, the optimal combination of prediction horizon N_p and control horizon N_c varies with operating conditions; thus, a uniformly distributed initial population is essential for thoroughly exploring the feasible domain. The Tent map offers good uniformity and ergodicity, ensuring population diversity from the outset and increasing the likelihood of identifying the globally optimal parameters.

Dynamic disturbance factor in the discoverer stage (Page 11, Section 3.2.1): The standard SSA frequently suffers from an imbalance between global exploration and local exploitation when addressing high-dimensional or constrained optimization problems. MPC parameter tuning requires both extensive exploration (to accommodate varying road conditions) and fine-grained local search (to achieve accurate tracking). The dynamic disturbance factor decreases with the number of iterations, enabling discoverers to conduct wide-ranging exploration in the early stages and gradually shift to refined local search later, thereby effectively balancing search diversity and convergence speed.

Cauchy mutation in the follower stage (Page 12, Section 3.2.2): The standard SSA is prone to becoming trapped in local optima when the population converges prematurely. In MPC parameter tuning, this may result in suboptimal control performance, particularly under

changing operating conditions. The Cauchy distribution has a long tail and generates larger perturbations than the Gaussian distribution, helping followers escape local optima. Combined with a greedy selection mechanism that retains only beneficial mutations, this approach enhances global search capability without sacrificing convergence.

Furthermore, we emphasize that these mechanisms are not arbitrary heuristics but are deliberately designed to address the practical demands of MPC parameter tuning—namely, adaptability to varying operating conditions, avoidance of local optima, and the balance between exploration and exploitation. The corresponding revisions have been incorporated into Section 3.2 of the revised manuscript.

2.1.2 Response to Comment 2

Comment 2：

The paper states that ISSA is used to periodically optimize the weight parameters of the MPC cost function, but the integration between the optimization algorithm and the MPC controller is not fully clarified. In particular, the manuscript does not clearly describe how frequently the optimization is executed, how the optimization variables are defined, and how the updated parameters influence the control process over time. A clearer explanation of the interaction between ISSA and the MPC control loop would improve the transparency and reproducibility of the proposed framework.

Response 2：

Thank you for your valuable comment. Regarding the integration between ISSA and the MPC controller, we have revised the manuscript to clarify this issue without adding a new section or introducing new parameter definitions. Specifically, we have added narrative descriptions in Section 3.1 and Section 3.3 to illustrate how ISSA interacts with the MPC control loop. The main additions are as follows:

Optimization frequency and triggering mechanism (Page 10, Section 3.1). We have clarified that the ISSA optimization is executed periodically (every 50 control cycles, approximately every 2 seconds) and can also be triggered on demand when a significant tracking error is detected.

Interaction mechanism between ISSA and MPC (Page 13, Section 3.3). We have described that ISSA runs as a background process that periodically updates the MPC weight parameters, and the updated parameters are subsequently used by the MPC controller in the following control cycles until the next optimization is triggered.

These revisions provide a clear explanation of how the ISSA-based weight adaptation is integrated with the MPC controller, addressing the reviewer’s concern without altering the original structure of the manuscript.

2.1.3 Response to Comment 3

Comment 3：

The AGV dynamic model is derived based on several simplifying assumptions, such as

small slip angles, planar motion, and simplified tire force relationships. While such assumptions are common in vehicle modeling, the manuscript does not sufficiently discuss their potential impact on control accuracy or the validity of the model under different operating conditions. A deeper discussion of the modeling limitations and their implications for the proposed control strategy would improve the technical rigor of the study.

Response 3：

Thank you for your comment regarding the modeling assumptions in the vehicle dynamics model. In response, we have added a brief discussion in Section 1.2 (Pages 6 and 7) to address the limitations of these assumptions and their impact on the control strategy. The main additions include: (1) clarifying the modeling assumptions (small slip angles, planar motion, simplified tire forces); (2) noting the potential model–plant mismatches that may arise under operating conditions such as sharp turns or load variations; and (3) highlighting that the ISSA-MPC framework offers inherent robustness against such model inaccuracies through its online adaptive optimization, as also verified by the experimental results presented in Section 4.3.

We believe these additions appropriately address the reviewer's concern.

2.1.4 Response to Comment 4

Comment 4：

The manuscript frequently claims that the improved algorithm enhances global exploration capability, prevents premature convergence, and improves tracking performance. However, these claims are primarily explained qualitatively. The paper would benefit from a more analytical discussion of how the algorithm modifications influence optimization behavior and why these changes are expected to produce better parameter tuning results for MPC.

Response 4：

Thank you for the insightful comment regarding the qualitative nature of the performance claims for the improved algorithm. We agree that a more analytical discussion of how the algorithmic improvements affect the optimization behavior would strengthen the persuasiveness of the manuscript. To address this concern, we have added analytical explanations in Sections 3.2, 3.2.1, 3.2.2, and 3.4.

The specific revisions are as follows:

Section 3.2 (Page 11): We added an introductory paragraph that explicitly states the three limitations of the standard SSA—uneven population distribution, imbalance between exploration and exploitation, and susceptibility to premature convergence—and explains how the three proposed improvement mechanisms (Tent chaotic initialization, dynamic disturbance factor, and Cauchy mutation) address these limitations in the context of MPC parameter tuning.

Section 3.2.1 (Page 11): We added an analytical explanation of how the dynamic disturbance factor balances global exploration and local exploitation. This is linked to the MPC parameter tuning problem, noting that the optimal weight combination varies with path

curvature or vehicle speed, and that the decreasing disturbance factor enables discoverers to perform coarse exploration initially and gradually shift to refined local search.

Section 3.2.2 (Page 12): We added analysis explaining why Cauchy mutation is more effective than Gaussian mutation for escaping local optima, emphasizing its long-tail distribution characteristics and the importance of avoiding suboptimal weight combinations for tracking performance.

Section 3.4 (Page 15): We added a paragraph explaining why the uniform distribution provided by Tent chaotic initialization is particularly advantageous for MPC parameter tuning—it increases the probability of covering the feasible search space from the initial stage, reducing the risk of converging to suboptimal solutions due to poor initialization.

We believe these additions provide the requested analytical depth and directly address the reviewer's concern.

2.1.5 Response to Comment 5

Comment 5:

Although the introduction reviews several trajectory tracking control approaches, the manuscript does not clearly position the proposed method within the broader research landscape of adaptive MPC and intelligent optimization-based controller tuning. Many existing studies have explored similar strategies that combine metaheuristic algorithms with MPC. The authors should more clearly highlight what differentiates their approach from existing methods and clarify the specific technical gap that this work aims to address.

Response 5:

Thank you for your comment regarding the positioning of the proposed method within the research context of adaptive MPC and intelligent optimization-based controller tuning. We agree that a clearer articulation of the technical gap and the distinctions from existing methods is essential.

To this end, we have revised the Section 0 Introduction by explicitly categorizing existing adaptive MPC approaches into two classes: model-based adaptation and parameter-tuning-based adaptation (Page 2). We note that although metaheuristic-based methods have been explored for offline MPC parameter optimization, they typically suffer from three limitations when applied to forklift-type AGVs under varying operating conditions: (1) an imbalance between global exploration and local exploitation, leading to suboptimal parameter sets; (2) susceptibility to premature convergence; and (3) a lack of online adaptation capability.

To highlight the differentiation of our work, we explicitly state that the proposed ISSA-MPC framework addresses these issues through three complementary mechanisms—Tent chaotic initialization, a dynamic disturbance factor, and Cauchy mutation—and employs periodic online optimization to achieve real-time adaptation to changing operating conditions (Page 3).

2.1.6 Response to Comment 6

Comment 6:

The background should give a more detailed literature review on the path-tracking of the AGV. Some recent work, such as "A Polytopic Model-based Robust Predictive Control Scheme for Path Tracking of Autonomous Vehicles, IEEE Transactions on Intelligent Vehicles, vol. 9, no. 2, pp. 3928-3939, Feb. 2024" and "ETS-Based Human–Machine Robust Shared Control Design Considering the Network Delays, IEEE Transactions on Automation Science and Engineering, vol. 22, pp. 17501-17511, 2025" can be referred.

Response 6:

Thank you for your suggestion to enrich the literature review on AGV path tracking and to incorporate recent relevant studies. We carefully considered the two recommended papers and have added them to the Section 0 Introduction to provide a more comprehensive and up-to-date review.

Specifically:

We added the polytopic model-based robust predictive control scheme ([25]) as a representative work on model-based adaptation, highlighting its contribution to path tracking robustness (Page 2).

We also added the ETS-based human–machine robust shared control design ([26]) as an example of another advanced robust control strategy that accounts for network delays, further demonstrating the breadth of recent developments in this field (Page 2).

These additions are placed within the section where we categorize existing adaptive MPC approaches, thereby strengthening the background literature and demonstrating that we have considered the most relevant recent studies. The corresponding references have been included in the reference list.

2.2 Comments from Reviewer #2

2.2.1 Response to Comment 1

Comment 1:

While the introduction effectively references traditional heuristic optimization methods such as Particle Swarm Optimization (PSO) and Ant Colony Optimization, the literature review feels slightly dated.

There is no mention of more recent and highly relevant trends in MPC parameter tuning and predictive optimization.

The authors must expand their introduction to discuss current state-of-the-art approaches, such as Reinforcement Learning (RL), Event-Triggered Hybrid Optimization, and the Alternating Direction Method of Multipliers (ADMM).

The authors should briefly contrast these modern techniques with their proposed ISSA

method to better justify why ISSA was chosen over these newer alternatives.

Response 1：

Thank you for the constructive comment regarding the literature review. We agree that the Introduction should reflect recent advances in MPC parameter tuning and predictive optimization to better position this work within the current research context.

To address this, we have revised the Section 0 Introduction as follows:

We added a concise discussion of three modern approaches: Reinforcement Learning (RL), event-triggered hybrid optimization, and the Alternating Direction Method of Multipliers (ADMM). For each method, we briefly summarized its advantages and limitations in the context of AGV trajectory tracking (Page 2).

We then compared these methods with the proposed ISSA-MPC framework, highlighting its advantages: lightweight structure, no requirement for pre-training, intuitive interpretability, and strong real-time adaptability under varying operating conditions (Page 3).

The corresponding references have been added to the reference list ([35], [36], [37] on Page 26).

2.2.2 Response to Comment 2

Comment 2：

The kinematic and dynamic models presented in Section 2 (Equations 1 through 13) rely on standard, well-established approaches for car-like and forklift-type mobile robots.

Because these models do not represent a novel contribution of this work, the step-by-step derivations are unnecessary and disrupt the flow of the manuscript.

I highly recommend that the authors summarize these sections. The authors should present only the final state-space equations utilized by the MPC and directly cite existing literature where the full derivations of these similar models have already been introduced.

Response 2：

Thank you for your valuable comments on Chapter 2. We fully agree that the kinematic and dynamic models themselves are not the main innovation of this paper. However, we respectfully request that the step-by-step derivation be retained for the following reasons:

Self-consistency: The derivation establishes the foundation for the subsequent MPC controller design, ISSA optimization, and simulation validation. Retaining the derivation ensures that the paper is self-contained, allowing readers to understand the entire design process without consulting external references.

Clarity of modeling assumptions: The derivation explicitly lists and justifies the key assumptions (small slip angles, planar motion, simplified tire forces, etc.). These assumptions are essential for understanding the scope and limitations of the model, particularly in the revised Section 1.2 where we specifically discuss their impact on control accuracy (Page 7). Removing

the derivation would leave this discussion without sufficient context.

Specificity of the forklift-type AGV: Compared to standard vehicle models, the forklift-type AGV has a unique structure (single steering wheel configuration, reference point selected at the fork end, etc.). A complete derivation helps clearly present these specific considerations, ensuring that the model accurately reflects the actual physical configuration of the experimental platform.

We understand the reviewer's concern regarding the conciseness of the manuscript. To address this, we have ensured that the derivation is presented succinctly and that the key equations are prominently highlighted.

2.2.3 Response to Comment 3

Comment 3：

The 3-DOF dynamic model relies heavily on the assumption of small wheel slip angles and neglects longitudinal-lateral coupling. While this holds true for the low-speed real-vehicle test (0.5 m/s), the authors must discuss the boundary limits of these assumptions.

If the AGV is subjected to high-speed cornering or heavy load transfers, how does the simplified linear tire model impact the predictive accuracy of the MPC?

Response 3：

Thank you for your expert comments regarding the assumptions of the 3-DOF dynamics model. We agree that it is important to discuss the boundary conditions of the linear tire model and its impact on prediction accuracy under more extreme operating conditions.

To address this, we have added the following discussion in Section 1.2 (Page 7):

"It should be noted that the vehicle dynamics model in this paper is derived based on several simplifying assumptions, including small slip angles, planar motion, and simplified tire force relationships. Under operating conditions such as sharp turns or significant load variations, the actual vehicle behavior may deviate from these assumptions, potentially leading to model–plant mismatches. Therefore, it is particularly important to clarify the applicable operating range of the linear tire model assumptions. In this study, the maximum speed of the forklift-type AGV is approximately 1.5 m/s, which aligns with typical factory floor operations. During indoor navigation, the speed is generally limited to within 1 m/s. Within this speed range, the wheel slip angles typically remain below 3–5°, which falls within the linear region of typical tire cornering stiffness characteristics. Consequently, the linear tire model provides sufficient tracking control accuracy under the tested conditions. In addition, the proposed ISSA-MPC framework offers inherent robustness against such modeling inaccuracies. By periodically optimizing the MPC weight parameters online based on real-time tracking errors, the controller can adapt to unmodeled dynamics without requiring an explicit model update."

This addition clarifies the applicable operating range of the simplified model (speed ≤ 1.5 m/s, slip angles ≤ 3–5°) and confirms that the experimental validation in this study (variable-speed real-vehicle tests and the limit simulation at 2 m/s) falls within the validity range of these

assumptions. Furthermore, we emphasize that the ISSA-MPC framework provides additional robustness against modeling inaccuracies through its online adaptive optimization mechanism.

2.2.4 Response to Comment 4

Comment 4：

Model Predictive Control is computationally demanding due to its rolling optimization mechanism. Integrating a population-based heuristic algorithm (ISSA) to optimize these parameters online during every control cycle introduces massive computational overhead.

The authors must explicitly state the control cycle time (sampling period T) and provide quantitative data on the average and peak execution times of the AT-ISSA-MPC algorithm. If the computational burden causes delays that exceed the control cycle, the stability of the AGV is compromised.

Response 4：

Thank you for the reviewer's insightful comment. In the revised manuscript, we have explicitly supplemented the control cycle and computation time data to address the concerns regarding computational burden and real-time performance.

In the real-vehicle experiments, the control cycle T was set to 0.04 s (25 Hz). The ISSA optimization runs as a background process and is triggered every 50 control cycles (i.e., every 2 s), rather than at every control cycle. The measured average execution time of the AT-ISSA-MPC algorithm was 28.5ms, with a peak execution time of 35.6ms, both of which are below the 40ms control cycle, leaving a sufficient safety margin. No control delays or stability issues were observed during the experiments.

These data demonstrate that the proposed method satisfies the real-time constraints on the embedded hardware platform and does not compromise the operational stability of the AGV due to computational load. The corresponding supplementary content has been added to Section 4.3 (Page 19).

2.2.5 Response to Comment 5

Comment 5：

In Section 3.1, the authors linearize the nonlinear system using a Taylor series expansion and discretize it using the forward Euler method.

Because the forward Euler method is a first-order approximation, its accuracy is heavily dependent on the chosen sampling period, T. The authors do not specify the exact value of T used in their simulations or real-vehicle experiments.

The authors must state the value of T and discuss the upper limit of this time step that ensures the linearization error remains bounded and does not degrade the predictive accuracy of the MPC.

The authors should discuss the trade-off here: a smaller T improves accuracy but

exponentially increases the computational load of the AT-ISSA-MPC algorithm. How did the authors balance this trade-off in their physical implementation?

Response 5：

Thank you for the reviewer's valuable comment. We note that the sampling period T = 40ms (control frequency 25 Hz) was already specified in Section 3.1 (Page 10)—we stated that the ISSA optimization is triggered every 50 control cycles at a control frequency of 25 Hz, from which T = 40ms can be derived.

However, we agree that the trade-off between discretization accuracy and computational load could be more explicitly connected. To address this, we added a sentence in Section 4.3 that directly links the measured execution times (28.5ms average, 35.6ms peak) to the selected T = 40ms, confirming that the balance between model fidelity and real-time performance is properly managed (Page 19). We believe these revisions adequately address the reviewer's concern.

2.2.6 Response to Comment 6

Comment 6：

（1）The authors assume the AGV operates "only on a planar surface, neglecting vertical motion". Warehouses and industrial floors frequently feature ramps, uneven transitions, and slight gradients. The authors must justify this assumption or discuss how unmodeled pitch dynamics/ramp traversal would affect the controller's stability.

（2）The dynamic model assumes "both the front-wheel steering angle and the wheel slip angles remain within a small range". Forklift-type AGVs frequently perform sharp maneuvers in tight aisles, involving large steering angles where the linear tire model breaks down. The authors must quantitatively define what this "small range" is (< 5 degrees) and explain how the MPC maintains tracking accuracy during aggressive, large-angle cornering.

（3）In Section 4.1, the authors assert that "Traditional parameter adjustment methods suffer from issues such as poor real-time performance and a tendency to get trapped in local optima". However, this claim is presented entirely without justification or supporting references. The authors must provide appropriate citations to substantiate this statement. Furthermore, they should explicitly define which specific "traditional methods" they are referring to (e.g., manual trial-and-error, basic grid search, or specific early metaheuristics), as the current phrasing is too vague.

（4）In Section 5, the authors state that "The double lane change maneuver and the straight-line deviation correction scenario are selected for simulation". However, the manuscript lacks a formal definition of these maneuvers. Additionally, as the double lane change is traditionally a high-speed automotive handling test, the authors should briefly justify its relevance for a forklift AGV operating at low speeds in a warehouse context.

Response 6：

Thank you for the reviewer's important comments.

Response to Comment (1):

We agree that in general industrial environments, ramps or uneven transitions may exist, and the influence of vertical motion should be considered when applying the controller to such scenarios. In this study, the planar assumption was adopted based on the following considerations:

① The experimental validation was conducted on an epoxy resin factory floor, which features minimal slopes and well-leveled surfaces, representing a common operational scenario for forklift-type AGVs in indoor logistics and manufacturing environments.

② The AGV operates at low speeds (≤1.5 m/s), under which the pitch dynamics induced by minor surface irregularities are negligible compared to the lateral and yaw dynamics that dominate trajectory tracking performance.

More importantly, the proposed ISSA-MPC framework inherently provides robustness against unmodeled disturbances—including minor pitch motions or gradual slopes—through its online weight adaptation mechanism, which adjusts control weights based on real-time tracking errors. The experimental results in Section 4.3 demonstrate satisfactory tracking performance under real operating conditions, indirectly confirming that any unmodeled effects remained within the controller's robustness margin. We also acknowledge that for more challenging terrains (e.g., steep ramps or speed bumps), the planar assumption may require re-examination, and we have noted this as a direction for future work.

Response to Comment (2):

We agree that the linear tire model assumption requires a clear quantitative definition, and that the controller's behavior under large steering angles should be addressed.

Quantitative definition of the "small range". In this study, the linear tire model is applied under the condition that both the front-wheel steering angle and wheel slip angles remain within 3–5°. This range is consistent with the linear region of typical tire cornering stiffness characteristics and is commonly adopted in low-speed AGV applications. Within this range, the linear approximation provides sufficient accuracy for trajectory tracking control.

Justification based on operating conditions. The forklift-type AGV in this study operates at speeds ≤1.5 m/s (typically ≤1.0 m/s during indoor navigation). Under such low-speed conditions, the required steering angles for trajectory tracking—even during relatively sharp turns in narrow aisles—generally fall within the 3–5° range when the path curvature is properly planned. The test scenarios (double lane change and straight-line deviation correction) reflect these typical operating conditions.

Robustness of the ISSA-MPC framework. For maneuvers that may temporarily exceed this linear range, the proposed ISSA-MPC controller provides robustness through its online weight adaptation mechanism. By periodically optimizing the MPC weight parameters based

on real-time tracking errors, the controller can compensate for model–plant mismatches caused by temporary nonlinear tire behavior. The experimental results in Section 4.3 demonstrate satisfactory performance under real factory conditions, indirectly validating the controller's robustness.

To address the reviewer's concern, we have added a concise explanation in Section 1.2 (Page 7). We also acknowledge that for extreme maneuvers involving sustained large steering angles, a nonlinear tire model may be required, and we have noted this as a direction for future work.

Response to Comment (3):

We agree that the original statement—"Traditional parameter adjustment methods suffer from issues such as poor real-time performance and a tendency to get trapped in local optima"—lacks supporting references and a clear definition of what constitutes "traditional methods."

To address this, we have revised the text in Section 3.2 (Page 10) as follows:

Clarify the scope of "traditional methods." We now explicitly distinguish between manual tuning methods (e.g., trial-and-error, grid search) and conventional metaheuristic algorithms used for offline tuning (e.g., standard PSO, GA).

Add supporting references. We have cited existing references from our bibliography to substantiate the claims. Specifically:

[15] (Li et al., 2025) and [31] (Jiang et al., 2021) support the limitations of manual trial-and-error methods in terms of efficiency and real-time applicability.

[30] (Wang et al., 2020) and [31] (Jiang et al., 2021) highlight the tendency of conventional metaheuristic algorithms (such as PSO and GA) to suffer from premature convergence to local optima in complex optimization tasks.

The revised text now reads:

"Traditional parameter adjustment methods—such as manual trial-and-error and basic grid search—suffer from poor real-time performance when applied to online MPC weight adaptation, as they lack efficient search mechanisms [15,31]. Meanwhile, conventional metaheuristic algorithms used for offline tuning (e.g., standard PSO or GA) are prone to premature convergence to local optima in complex, high-dimensional search spaces [30,31]. Hence, intelligent algorithms with enhanced global search capability are frequently employed to optimize these parameters."

Response to Comment (4):

We agree that the original manuscript lacks formal definitions of the simulation maneuvers and does not justify the relevance of the double lane change scenario for a low-speed forklift

AGV.

To address this, we have added definitions for both maneuvers in Section 4.1 (Pages 15 and 16) and Section 4.2 (Page 18):

Double lane change maneuver: This scenario originates from automotive handling tests but is adopted here as a representative case of continuous curvature variation, allowing rigorous evaluation of the controller's dynamic tracking performance under lateral maneuvers that mimic obstacle avoidance and aisle turning in warehouse environments.

Straight-line deviation correction scenario: This scenario is designed to evaluate the controller's convergence performance under an initial lateral offset. The reference path is a straight line, and the vehicle starts with an initial lateral error of 0.5 m without an initial heading error.

Regarding the relevance of the double lane change maneuver for a low-speed AGV, we note that although this maneuver is traditionally used for high-speed automotive testing, its continuous curvature variation makes it an ideal benchmark for evaluating the dynamic response of trajectory tracking controllers. For forklift AGVs, warehouse scenarios such as turning, obstacle avoidance, and narrow-aisle navigation also involve continuous curvature changes. Conducting this test at low speed (2 m/s) provides a rigorous assessment of the controller's lateral tracking capability under varying curvature, and the findings are generalizable to similar real-world scenarios.

2.2.7 Response to Comment 7

Comment 7：

（1）While the authors correctly identify that the AT-ISSA-MPC yields lower peak and root mean square errors compared to the FT-MPC in the double lane change maneuver, the commentary surrounding these graphs (Figures 6-9) is insufficient. The authors merely report the numerical improvements without providing a control-theoretic or physical explanation for the results. The authors must discuss why the tracking errors peak at specific locations along the trajectory (e.g., at the points of maximum curvature).

（2）The authors must explicitly explain the mechanism of improvement. Why exactly does the fixed time-domain (FT-MPC) struggle at these trajectory inflection points, and how precisely does the adaptive adjustment of the prediction and control horizons (Np and Nc) by the ISSA algorithm mitigate this?

（3）The simulation is currently only performed at a single constant speed of v = 2 m/s. The primary advantage of an adaptive parameter strategy should be its robustness to varying or extreme dynamic conditions. To truly demonstrate the necessity and superiority of the AT-ISSA-MPC approach, the authors should conduct and discuss simulations across different operating conditions—most notably at higher speeds, where dynamic constraints are tighter and the FT-MPC is more likely to fail.

（4）In the straight-line deviation scenario (Figure 10) , the authors highlight the

improved longitudinal convergence distance (20m vs. 28m). However, they ignore the transient behavior. The FT-MPC clearly exhibits a lateral overshoot before converging on the reference trajectory. The authors must explicitly quantify and discuss this overshoot and how the AT-ISSA-MPC improves the damping of the system.

Response 7：

Thank you for the reviewer’s important comments.

Response to Comment (1):

We agree that the initial analysis of the double lane change results in Section 4.1 was primarily descriptive. To address this, we have revised and streamlined the analysis in Section 4.1 (Page 18), as detailed below:

Correlation with curvature. We now explicitly note that the lateral error and heading error both peak near the regions of maximum curvature. This is because high-curvature segments demand larger steering angles and lateral accelerations, posing greater challenges for trajectory tracking.

Mechanism of improvement. We compare the FT-MPC (which uses constant weights and inevitably represents a compromise between low- and high-curvature segments) with the AT-ISSA-MPC (which periodically optimizes weights online based on real-time tracking errors). When approaching a high-curvature segment, the increasing error triggers the optimizer to adjust the weights—typically by increasing the penalty on lateral deviation—thereby reducing the peak error.

Phase relationship. As observed in Figs. 7 and 9, the heading error peaks slightly after the lateral error. This order is physically plausible: when the vehicle enters a high-curvature region, lateral deviation develops first, followed by steering adjustments that subsequently induce heading deviation.

Concise numerical summary. The quantitative results are now presented in a condensed format, retaining all key error metrics and improvement data.

Response to Comment (2):

We thank the reviewer for this insightful comment. We agree that the current manuscript does not sufficiently explain why the fixed time-domain MPC (FT-MPC) struggles at trajectory inflection points and how the adaptive adjustment of the prediction horizon N_p and control horizon N_c by ISSA mitigates this issue. To address this, we have added the following analysis in Sections 3.2 and 4.1:

①Why FT-MPC struggles at inflection points.

At trajectory inflection points (regions of high curvature), the reference path changes

direction rapidly. For a fixed-horizon MPC, N_p and N_c are constant. If N_p is too short, the controller cannot anticipate the upcoming curvature change, leading to delayed steering response and increased tracking error. If N_p is too long, model prediction errors accumulate, causing the controller to react prematurely or oscillate. Similarly, a fixed N_c limits the controller's ability to adjust control inputs over an appropriate future window—too few steps may cause abrupt steering, while too many may lead to unnecessary control activity. Thus, a fixed parameter set represents a compromise that cannot simultaneously optimize performance across both low- and high-curvature segments.

②How ISSA adaptive adjustment mitigates this.

The ISSA algorithm periodically optimizes N_p and N_c online based on real-time tracking error feedback. When the vehicle approaches a high-curvature segment, the increasing lateral error triggers the optimizer to select a moderately longer prediction horizon (to anticipate the upcoming curve) paired with a properly sized control horizon (to enable smooth yet responsive steering adjustments). This adaptive strategy enables the controller to temporarily increase its "look-ahead" capability at curves while maintaining real-time responsiveness. After passing the inflection point, the optimizer reverts to shorter horizons suitable for straight-line tracking. This dynamic adjustment of N_p and N_c directly addresses the compromise inherent in fixed-horizon MPC.

③Supporting discussion added.

In Section 3.2 (Page 11), we added a concise explanation of how the ISSA optimizer balances N_p and N_c under varying curvature. In Section 4.1, we linked the observed error reduction (especially near curvature peaks) to this adaptive mechanism, noting that the AT-ISSA-MPC achieves lower peak errors because it "extends its prediction horizon when approaching curves and shortens it after exiting" (Page 18).

Response to Comment (3):

We agree that evaluating the proposed AT-ISSA-MPC under varying speed conditions is important to demonstrate its robustness and necessity.

While the simulation in Section 4.1 was performed at a constant speed of 2 m/s for controlled comparison, the real-vehicle experiments in Section 4.3 already include variable-speed operation. As described in Section 4.3, the forklift AGV operated at 0.5 m/s in curved segments and 1 m/s in straight segments, with speed transitions throughout the test. The results (Figs. 12-13, Table 3) show that AT-ISSA-MPC consistently outperforms FT-MPC under these varying-speed conditions, achieving a 49.37% reduction in peak lateral error and a 40.13% reduction in mean square error.

This variable-speed experimental validation demonstrates that the proposed method maintains its advantage even when operating speeds change, confirming its practical robustness. We have noted this in Section 4.3.

For future work, we plan to extend the simulation study to cover a wider range of speeds (including higher speeds beyond the current operational limit) to further validate the method's performance envelope. This has been added as a direction for future research in the Conclusion section.

Response to Comment (4):

We agree that the analysis of the straight-line deviation scenario in Section 4.2 focused primarily on convergence distance and did not explicitly quantify the lateral overshoot exhibited by FT-MPC in Fig. 10.

As observed in Fig. 10, the FT-MPC exhibits a noticeable lateral overshoot before converging to the reference trajectory, while the AT-ISSA-MPC shows significantly smaller overshoot and faster damping. This transient behavior is consistent with the control mechanism discussed in Sections 3.2 and 4.1: the FT-MPC employs fixed weight parameters and fixed control horizons, which represent a compromise that may lead to overcorrection during initial error recovery. In contrast, the AT-ISSA-MPC adaptively optimizes the MPC weight parameters (particularly the lateral error penalty) and the time-domain parameters N_p and N_c based on real-time tracking error, enabling a more appropriately damped response that avoids overshoot while maintaining rapid convergence.

The improved damping observed in Fig. 10 directly supports the effectiveness of the proposed adaptive strategy. We have noted this in the existing figure caption, which states that AT-ISSA-MPC "has a smaller overshoot and converges more quickly."

2.2.8 Response to Comment 8

Comment 8:

The experimental validation section (Section 5.3) lacks rigorous description and exhibits significant discrepancies when compared to the simulation section:

(1) The authors do not properly describe the trajectory used for the physical experiment (Figure 12). Why was a simple diagonal trajectory selected for the real-world test instead of replicating the simulation trajectories (double lane change and straight-line deviation)? Replicating the simulation paths in the real world is crucial to directly validate the simulation model's fidelity. Furthermore, the authors must discuss how the physical controller would perform under more complex, realistic trajectories (e.g., 90-degree turns, U-turns, or navigating around obstacles).

(2) The simulations were conducted at 2 m/s , while the real-vehicle experiments were limited to 0.5 m/s. The authors state this was for "safety", but this drastic reduction in speed inherently reduces the dynamic complexity of the system. Operating at 0.5 m/s minimizes slip angles and inertial forces, which might render the complex AT-ISSA-MPC unnecessary compared to a simpler controller. The authors must justify this discrepancy and discuss whether the physical hardware could handle the algorithm at higher speeds.

Response 8:

Thank you for the reviewer’s important comments.

Response to Comment (1):

The trajectory selected for the real-vehicle experiments is a straight path with a 135° turn, as shown in Fig. 12 (Page 20). This trajectory was chosen primarily for safety considerations. In the initial phase of the real-vehicle experiments, our top priority was to ensure the safety of the platform and the surrounding environment. Selecting a simple trajectory with a single turn allowed us to systematically evaluate the tracking performance of the proposed controller under controlled conditions, laying the foundation for subsequent tests on more complex trajectories.

Response to Comment (2):

We provide the following explanation and justification for the speed difference between simulation and real-vehicle experiments:

①Typical operating speed of the forklift AGV: As noted in our response regarding the modeling assumptions, the maximum speed of the forklift-type AGV used in this study is approximately 1.5 m/s, which aligns with typical factory floor operations. In indoor factory environments, AGVs generally reduce speed when navigating narrow aisles and making turns to ensure safety and accuracy. Therefore, the speed settings of 1 m/s on straight segments and 0.5 m/s on curved segments reflect actual operating conditions rather than artificial limitations.

②Safety considerations in the initial validation phase: The real-vehicle experiments were conducted in a factory environment with limited space and the potential presence of unexpected obstacles. Conservative speed selection was necessary to ensure the safety of personnel and equipment.

③Dynamic complexity at 0.5 m/s versus 2 m/s: We agree that operation at 0.5 m/s reduces slip angles and inertial forces, potentially simplifying the tracking problem. However, it is important to note that even at 0.5 m/s, the forklift AGV remains subject to nonholonomic constraints, requiring coordinated steering and driving control. More importantly, the simulation results at 2 m/s demonstrate that the proposed ISSA-MPC algorithm achieves significant improvements over the baseline MPC under higher dynamic complexity. This indicates that the advantages of the proposed method are not limited to low-speed conditions.

④Hardware capability at higher speeds: Regarding the reviewer’s concern about hardware limitations, we have conducted preliminary tests on straight segments at 1 m/s. The by-wire chassis and the onboard computing unit (industrial computer) were able to execute the ISSA-MPC algorithm within the required control cycle (25 Hz) without noticeable delays or stability issues. Relevant discussion has been added to Section 4.3 (Page 19). In future work, we plan to systematically test at higher speeds to further validate the robustness of the algorithm.

2.2.9 Response to Comment 9

Comment 9：

In Section 5.3, the authors highlight that the AT-ISSA-MPC reduced the mean lateral displacement error to 0.0108m compared to the FT-MPC's 0.0252m. However, as both algorithms successfully guide the AGV to its final destination, the authors fail to explain the practical significance of this improvement. The authors must explicitly comment on why minimizing intermediate trajectory error is critical in this context. For example, how do these error margins relate to physical constraints such as narrow warehouse aisles, standard forklift operating clearances, or collision avoidance?

Response 9：

Thank you for this insightful comment. We agree that merely reporting numerical error reductions is insufficient to illustrate their practical significance for forklift-type AGV applications. To address this, we have added relevant discussion in Section 4.3 (Page 21), clarifying the following points:

①Narrow warehouse aisles and operational clearances: In typical factory environments, AGVs often operate in narrow aisles with limited clearance between the vehicle and surrounding obstacles. Reducing the maximum lateral tracking error from 0.68704 m to 0.34785 m (a reduction of 49.37%) significantly increases the safety margin against collisions, effectively mitigating the risk of path deviation-related incidents.

②Error accumulation over multi-segment paths: Although both algorithms successfully guide the AGV to its destination, minimizing intermediate path errors prevents error accumulation that could lead to positioning failures at critical points such as turns or docking stations.

③Consistency with simulation results: The error improvement observed in the real-vehicle experiments (49.37% reduction in peak error) is highly consistent with the simulation results under the double lane change maneuver (improvements of 38–72%), confirming that the tracking performance enhancement achieved by the proposed method translates effectively from simulation to practical application scenarios.

Yours sincerely!

Corresponding author: Chengjun Ding

Email: dcj@hebut.edu.cn

April 4, 2026

School of Mechanical Engineering, Hebei University of Technology, Tianjin, China
