# Supplementary material for: Enhanced accuracy and adaptability: An ISSA-optimized MPC approach for AGV trajectory tracking
Source: PLoS One. 2026 May 21;21(5):e0345476. doi: 10.1371/journal.pone.0345476 (PMC13193454; doi:10.1371/journal.pone.0345476)
Supplement: Attachment — Submitted filename: Review.docx [file pone.0345476.s001.docx]

**Manuscript Number:** PONE-D-26-10662 **Title:** Enhanced Accuracy and Adaptability: An ISSA-Optimized MPC Approach for AGV Trajectory Tracking

The authors presented an adaptive trajectory tracking strategy for automated guided vehicles (AGVs) by combining Model Predictive Control (MPC) with an Improved Sparrow Search Algorithm (ISSA). They established a kinematic and a simplified three-degree-of-freedom (3-DOF) dynamic model for a forklift-type AGV. To overcome the limitations of fixed-weight MPC, the authors propose tuning the time-domain parameters online using ISSA, which is enhanced via Tent chaotic mapping for population initialization, dynamic disturbance factors, and Cauchy mutation operators to balance global and local searches. The proposed AT-ISSA-MPC controller is validated against a standard Fixed Time-domain MPC (FT-MPC) through ROS/Gazebo simulations and a real-world test on a forklift AGV operating at 0.5m/s.

Comment 1:

While the introduction effectively references traditional heuristic optimization methods such as Particle Swarm Optimization (PSO) and Ant Colony Optimization, the literature review feels slightly dated.

- There is no mention of more recent and highly relevant trends in MPC parameter tuning and predictive optimization.
- The authors must expand their introduction to discuss current state-of-the-art approaches, such as Reinforcement Learning (RL), Event-Triggered Hybrid Optimization, and the Alternating Direction Method of Multipliers (ADMM).
- The authors should briefly contrast these modern techniques with their proposed ISSA method to better justify why ISSA was chosen over these newer alternatives.

Comment 2:

The kinematic and dynamic models presented in Section 2 (Equations 1 through 13) rely on standard, well-established approaches for car-like and forklift-type mobile robots.

- Because these models do not represent a novel contribution of this work, the step-by-step derivations are unnecessary and disrupt the flow of the manuscript.
- I highly recommend that the authors summarize these sections. The authors should present only the final state-space equations utilized by the MPC and directly cite existing literature where the full derivations of these similar models have already been introduced.

Comment 3:

The 3-DOF dynamic model relies heavily on the assumption of small wheel slip angles and neglects longitudinal-lateral coupling. While this holds true for the low-speed real-vehicle test (0.5 m/s), the authors must discuss the boundary limits of these assumptions.

- If the AGV is subjected to high-speed cornering or heavy load transfers, how does the simplified linear tire model impact the predictive accuracy of the MPC?

Comment 4:

Model Predictive Control is computationally demanding due to its rolling optimization mechanism. Integrating a population-based heuristic algorithm (ISSA) to optimize these parameters *online* during every control cycle introduces massive computational overhead.

- The authors must explicitly state the control cycle time (sampling period T) and provide quantitative data on the average and peak execution times of the AT-ISSA-MPC algorithm. If the computational burden causes delays that exceed the control cycle, the stability of the AGV is compromised.

Comment 5:

In Section 3.1, the authors linearize the nonlinear system using a Taylor series expansion and discretize it using the forward Euler method.

- Because the forward Euler method is a first-order approximation, its accuracy is heavily dependent on the chosen sampling period, T. The authors do not specify the exact value of T used in their simulations or real-vehicle experiments.
- The authors must state the value of T and discuss the upper limit of this time step that ensures the linearization error remains bounded and does not degrade the predictive accuracy of the MPC.
- The authors should discuss the trade-off here: a smaller T improves accuracy but exponentially increases the computational load of the AT-ISSA-MPC algorithm. How did the authors balance this trade-off in their physical implementation?

Comment 6:

- The authors assume the AGV operates "only on a planar surface, neglecting vertical motion". Warehouses and industrial floors frequently feature ramps, uneven transitions, and slight gradients. The authors must justify this assumption or discuss how unmodeled pitch dynamics/ramp traversal would affect the controller's stability.
- The dynamic model assumes "both the front-wheel steering angle and the wheel slip angles remain within a small range". Forklift-type AGVs frequently perform sharp maneuvers in tight aisles, involving large steering angles where the linear tire model breaks down. The authors must quantitatively define what this "small range" is (< 5 degrees) and explain how the MPC maintains tracking accuracy during aggressive, large-angle cornering.
- In Section 4.1, the authors assert that "Traditional parameter adjustment methods suffer from issues such as poor real-time performance and a tendency to get trapped in local optima". However, this claim is presented entirely without justification or supporting references. The authors must provide appropriate citations to substantiate this statement. Furthermore, they should explicitly define which specific "traditional methods" they are referring to (e.g., manual trial-and-error, basic grid search, or specific early metaheuristics), as the current phrasing is too vague.
- In Section 5, the authors state that "The double lane change maneuver and the straight-line deviation correction scenario are selected for simulation". However, the manuscript lacks a formal definition of these maneuvers. Additionally, as the double lane change is traditionally a high-speed automotive handling test, the authors should briefly justify its relevance for a forklift AGV operating at low speeds in a warehouse context.

Comment 7:

While the authors correctly identify that the AT-ISSA-MPC yields lower peak and root mean square errors compared to the FT-MPC in the double lane change maneuver, the commentary surrounding these graphs (Figures 6-9) is insufficient.

- The authors merely report the numerical improvements without providing a control-theoretic or physical explanation for the results. The authors must discuss *why* the tracking errors peak at specific locations along the trajectory (e.g., at the points of maximum curvature).
- The authors must explicitly explain the mechanism of improvement. Why exactly does the fixed time-domain (FT-MPC) struggle at these trajectory inflection points, and how precisely does the adaptive adjustment of the prediction and control horizons (Np and Nc) by the ISSA algorithm mitigate this?
- The simulation is currently only performed at a single constant speed of v = 2 m/s. The primary advantage of an *adaptive* parameter strategy should be its robustness to varying or extreme dynamic conditions. To truly demonstrate the necessity and superiority of the AT-ISSA-MPC approach, the authors should conduct and discuss simulations across different operating conditions—most notably at higher speeds, where dynamic constraints are tighter and the FT-MPC is more likely to fail.
- In the straight-line deviation scenario (Figure 10) , the authors highlight the improved longitudinal convergence distance (20m vs. 28m). However, they ignore the transient behavior. The FT-MPC clearly exhibits a lateral overshoot before converging on the reference trajectory. The authors must explicitly quantify and discuss this overshoot and how the AT-ISSA-MPC improves the damping of the system.

Comment 8:

The experimental validation section (Section 5.3) lacks rigorous description and exhibits significant discrepancies when compared to the simulation section:

- The authors do not properly describe the trajectory used for the physical experiment (Figure 12). Why was a simple diagonal trajectory selected for the real-world test instead of replicating the simulation trajectories (double lane change and straight-line deviation)? Replicating the simulation paths in the real world is crucial to directly validate the simulation model's fidelity. Furthermore, the authors must discuss how the physical controller would perform under more complex, realistic trajectories (e.g., 90-degree turns, U-turns, or navigating around obstacles).
- The simulations were conducted at 2 m/s , while the real-vehicle experiments were limited to 0.5 m/s. The authors state this was for "safety", but this drastic reduction in speed inherently reduces the dynamic complexity of the system. Operating at 0.5 m/s minimizes slip angles and inertial forces, which might render the complex AT-ISSA-MPC unnecessary compared to a simpler controller. The authors must justify this discrepancy and discuss whether the physical hardware could handle the algorithm at higher speeds.

Comment 9:

In Section 5.3, the authors highlight that the AT-ISSA-MPC reduced the mean lateral displacement error to 0.0108m compared to the FT-MPC's 0.0252m. However, as both algorithms successfully guide the AGV to its final destination, the authors fail to explain the practical significance of this improvement. The authors must explicitly comment on *why* minimizing intermediate trajectory error is critical in this context. For example, how do these error margins relate to physical constraints such as narrow warehouse aisles, standard forklift operating clearances, or collision avoidance?
